# Supplementary material for: A Critical View of the Application of the APEX Software (Aqueous Photochemistry of Environmentally-Occurring Xenobiotics) to Predict Photoreaction Kinetics in Surface Freshwaters
Source: Molecules. 2019 Dec 18;25(1):9. doi: 10.3390/molecules25010009 (PMC7017383; doi:10.3390/molecules25010009)
Supplement: Supplementary file 1 [file molecules-25-00009-s001.zip › 3.2.4_gcc-4.4.0.zip!/3.2.4_gcc-4.4.0/3.2.4_gcc-4.4.0/doc/octave/html/interpreter/Installing-EOS.html]

Installing EOS - Untitled


Next: Using Octave Mode,
Up: Emacs Octave Support


---

### G.1 Installing EOS

The Emacs package EOS consists of the three files octave-mod.el,
octave-inf.el, and octave-hlp.el. These files, or better
yet their byte-compiled versions, should be somewhere in your Emacs
load-path.

If you have GNU Emacs with a version number at least as high as 19.35,
you are all set up, because EOS is respectively will be part of GNU
Emacs as of version 19.35.

Otherwise, copy the three files from the emacs subdirectory of
the Octave distribution to a place where Emacs can find them (this
depends on how your Emacs was installed). Byte-compile them for speed
if you want.
